# The emerging role of super enhancer-derived noncoding RNAs in human cancer

**DOI:** 10.7150/thno.49168

**Published:** 2020-09-02

**Authors:** Yutong Wang, Hui Nie, Xiaoyun He, Zhiming Liao, Yangying Zhou, Jianhua Zhou, Chunlin Ou

**Affiliations:** 1 Department of Pathology, Xiangya Hospital, Central South University, Changsha, Hunan, 410008, China.; 2 Department of Endocrinology, Xiangya Hospital, Central South University, Changsha, Hunan 410008, China.; 3 Department of Oncology, Xiangya Hospital, Central South University, Changsha 410008, Hunan, China.; 4 National Clinical Research Center for Geriatric Disorders, Xiangya Hospital, Central South University, Changsha 410008, Hunan, China.

**Keywords:** Super enhancers, Noncoding RNAs, Tumorigenesis, Inflammatory response, Therapy

## Abstract

Super enhancers (SEs) are large clusters of adjacent enhancers that drive the expression of genes which regulate cellular identity; SE regions can be enriched with a high density of transcription factors, co-factors, and enhancer-associated epigenetic modifications. Through enhanced activation of their target genes, SEs play an important role in various diseases and conditions, including cancer. Recent studies have shown that SEs not only activate the transcriptional expression of coding genes to directly regulate biological functions, but also drive the transcriptional expression of non-coding RNAs (ncRNAs) to indirectly regulate biological functions. SE-derived ncRNAs play critical roles in tumorigenesis, including malignant proliferation, metastasis, drug resistance, and inflammatory response. Moreover, the abnormal expression of SE-derived ncRNAs is closely related to the clinical and pathological characterization of tumors. In this review, we summarize the functions and roles of SE-derived ncRNAs in tumorigenesis and discuss their prospective applications in tumor therapy. A deeper understanding of the potential mechanism underlying the action of SE-derived ncRNAs in tumorigenesis may provide new strategies for the early diagnosis of tumors and targeted therapy.

## Introduction

A tumor is a malignant mass of cancerous cells that undergoes uncontrolled growth and replication. Developing tumors invade surrounding tissues and cause organ failure. Although significant advances have been made in the understanding and treatment of tumors in recent years, the morbidity and mortality rates remain high due to tumor recurrence and metastasis 1. Therefore, to improve the overall survival of patients with cancer, early screening and tumor marker detection are important clinical strategies. Tumor biomarkers are molecules expressed in cancer cells and tissues that can reflect the progression and prognosis of malignant tumors 2. They can be divided into five groups according to their biological functions: (1) oncofetal proteins, (2) tumor antigens, (3) enzymes, (4) hormones, and (5) special plasma proteins. In recent years, studies have found that non-coding RNAs (ncRNAs) have potential tumor marker characteristics. It is possible that analyzing ncRNAs may help diagnose many malignant tumors, such as gastric cancer, bladder cancer, prostate cancer, pancreatic cancer and cholangiocarcinoma 3-6.

With the advent of the post-genomic era, humans have been gaining a greater understanding of the genome and enhancers. In 2013, Young 7 proposed the concept of super enhancers (SEs). SEs are large clusters of adjacent enhancers that drive the expression of genes which regulate cellular identity; SE regions can be enriched with a high density of transcription factors (TFs), co-factors, and enhancer-associated epigenetic modifications. SEs can activate the expression of genes that determine cellular identity, thus affecting the occurrence of tumors and other diseases. It has been revealed in recent years that SEs not only directly activate exon-encoded genes to regulate biological functions, but they also activate the expression of ncRNAs, which regulate biological processes indirectly. In 2017, Suzuki *et al.* 8 reported that SEs are the core of tissue-specific miRNA networks and influence the progress of multiple tumors by regulating miRNA production. During the process of cell transformation, activated SEs usually regulate the generation of pro-carcinogenic miRNAs, and the inhibited SEs are often associated with tumor suppressor miRNAs. Therefore, the combination of SEs with multiple downstream miRNAs has the potential to serve as a cancer-related biomarker. The "SE-TF-ncRNA-target gene" regulatory networks have been widely studied. This review aims to describe and summarize several kinds of SE-derived ncRNAs and their functions in tumorigenesis, and primarily discuss their prospective clinical applications, aiming at providing new strategies for tumor-targeted therapies.

## Biogenesis and characteristics of SEs

**Figure 1:**
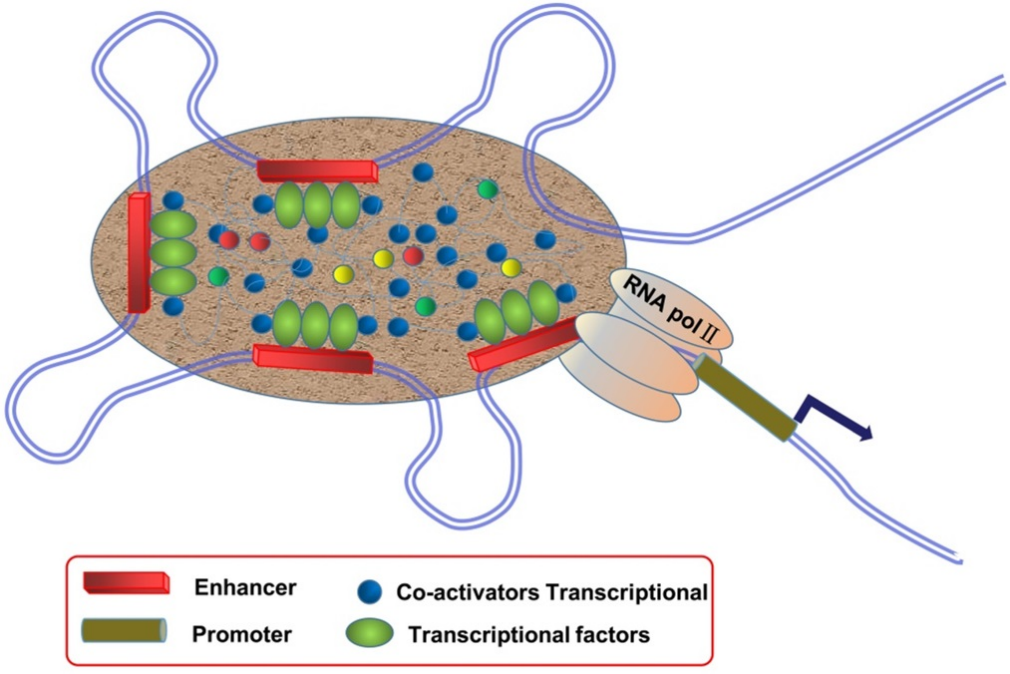
Schematic diagram of super enhancers (SEs). SEs are large clusters of adjacent enhancers that drive the expression of genes that regulate cellular identity, and SE regions can be enriched with a high density of transcription factors, co-factors, and enhancer-associated epigenetic modifications.

SEs are large clusters of adjacent enhancers, and SE regions can be enriched with a high density of TFs, co-factors, and enhancer-associated epigenetic modifications 9, 10 (**Figure 1**). Compared to typical enhancers (TEs), SEs have the following functional characteristics: (1) modified with a high density of H3K27ac and H3K4me1, as well as binding mediator complex and Bromodomain-containing protein 4 (BRD4); (2) binding with TFs and transcriptional activity-associated chromosome markers; (3) regulation of gene expression; and (4) sensitivity to the effect of transcriptional inhibitors 11-13. SEs exist in many cell types and are closely related to biological growth and cancer. They are usually enriched in genetic susceptibility regions of the genome, which are closely associated with a variety of disease pedigrees, suggesting that they may play an important role in disease diagnosis and treatment.

At present, SEs are identified based on enhancers, involving three steps: (1) identifying the active enhancer sites, (2) stitching the enhancers, and (3) determining the threshold between the SEs and normal enhancers. First, chromatin immunoprecipitation (ChIP-seq) is used to detect factors or histone modifications associated with active enhancers, such as TFs, transcriptional coactivators (*e.g.* Mediator, p300, etc.), and histone modifications H3K27ac and H3K4me1. Next, the obtained enhancers are stitched. Researchers concatenate the enhancers within 12.5 kb of each other to define a single entity called the “stitched enhancer.” Lastly, to determine the threshold, they rank the stitched enhancer entities and the remaining individual enhancers (those without a neighboring enhancer within 12.5 kb) based on the total background-normalized level of the Med1 signal within the genomic region to obtain a resultant curve. The signal value obtained at the tangential point of the line with slope 1 on the curve is used as the threshold to distinguish SEs from normal enhancers. SEs are defined as regions to the right of the threshold of the resulting curve, while normal enhancers are defined, as those to the left 14. With the development of next-generation sequencing technologies, more methods of SE identification have been used (**Table 1**). After identifying SEs, the expression of protein-coding genes and ncRNAs regulated by SEs can be predicted according to the gene location. Through RNA-seq, it is possible to establish a network between SEs and abnormally expressed mRNAs and ncRNAs. In addition, we can speculate on the key SEs and SE-derived ncRNAs in tumors. Information on SE-derived ncRNAs may provide a theoretical basis for future studies on the mechanisms underlying tumors and their treatment.

### Classification of SE-derived ncRNAs

SEs not only directly activate exon-encoded genes to regulate biological functions, but also activate the expression of ncRNAs, which regulate biological processes indirectly. These ncRNAs mainly include microRNAs (miRNAs), long non-coding RNAs (lncRNAs), circular RNAs (circRNAs), and enhancer RNAs (eRNAs) (**Table 2**). The activation of SEs can induce ncRNAs to regulate target genes in various ways. On one hand, they can promote the transcription and maturation of miRNAs, transcription and generation of lncRNAs, and transcription and ring formation of circRNAs. On the other hand, the transcription products of SEs themselves, that is eRNAs, also play a synergistic role in regulating gene expression. miRNAs were the first ncRNAs to be identified. After transcription, the initial product of a miRNA undergoes two shears, one in the nucleus and one in the cytoplasm, to form a mature miRNA 23, 24. Suzuki *et al.* 8 reported that SEs promote the maturity of pri-miRNAs by recruiting Drosha/DGCR 8 to regulate the transcription and synthesis of tissue-specific miRNAs (miR-290-295 and miR-106a-363). SE-derived lncRNAs usually have two forms, one of which is eRNA. An eRNA is an ncRNA formed after the self-transcription of the enhancer itself, with a sequence length of 0.5-5 kb; therefore, it is also classified as a lncRNA. There are many mechanisms by which eRNAs affect gene expression 25, 26. The other form is lncRNA, which are transcribed from the promoter region through the regulation of SEs; we normally call these SE-derived lncRNAs 27. Recent studies suggested that SE-derived lncRNAs can interact with exon-sensitive lncRNAs, and this interaction activates promoters and enhancers, thereby promoting chromatin loop integration and nuclear topological domain formation of the target genes, contributing to their expression 28. At present, more researchers are examining the mechanism underlying the action of SE-derived lncRNAs in tumors and other diseases. circRNAs are a class of recently discovered ncRNAs. Studies on the mechanism of the formation of SE-derived circRNAs and the role of SEs remain scarce. However, by using polymerase chain reaction (PCR) and *in situ* hybridization (ISH), scientists have detected the expression levels of circRNAs in various human tissues and found that SE-derived circRNAs have higher expression levels and stronger tissue specificity than TE-derived circRNAs 29.

## Biological functions of SE-derived ncRNAs

SE-derived ncRNAs are widely expressed in a variety of cells and are involved in various diseases, such as diabetes, rheumatoid arthritis, and tumors. SE-derived ncRNAs also play an important role in regulating the following biological activities via a series of action modes (**Table 2**).

### Determination of tissue specificity

Song *et al.* 38 pointed out that the methylation of enhancer DNA is closely related to the heterogeneity of cells. DNA methylation of Sox2 and Mir290 SEs can guide the differentiation of embryonic stem cells. Suzuki *et al.* 8 reported that SEs are highly correlated with miRNAs in tissue-specific determination. Several tissue-specific SE-derived miRNAs have been detected: miR-290-295 in mouse embryonic cells (mESCs), miR-142 in mouse precursor B cells, miR-1/133a2 in myotube tissues, and miR-142/210 in Th cells. It has been shown that these tissue-specific SE-derived miRNAs are part of a gene regulatory network that controls cell-type specificity, and tissue specificity may be attributed to target avoidance phenomena between miRNAs and target genes 39. Ounzain *et al.* 40 found a cardiomyocyte-specific SE-derived lncRNA, CARMEN. Research has shown that CARMEN can affect the fate of cardiomyocytes and interact with TFs SUZ12 and EZH2.

### Regulating growth and development

SE-derived ncRNAs play an important role in the growth and development of neonates. Ounzain *et al.* 41 reported that Novlnc6, an SE-derived lncRNA, is associated with cardiac development. Novlnc6 is regulated by many important major TFs and regulatory proteins in the heart, including TBX20 and MEF2A. Anderson *et al.* 42 reported that SE-derived lncRNAs upregulate cardiac development by interacting with the TF HAND2. Other studies found that the SE-derived lncRNA Whisper can induce myocardial remodeling 43, whereas SE-circNfix inhibits the repair and regeneration of mouse cardiomyocytes 29. SE-miR140 may cause skeletal dysplasia 44, and Degirmenci *et al.* 45 reported that the eRNA lncASIR is associated with insulin-induced adipocyte metabolism. The eRNA Bloodlnc can promote erythroblast proliferation and enucleation of red blood cells and participate in erythrocyte proliferation and differentiation 46.

### Regulating tumor progression

**Figure 2:**
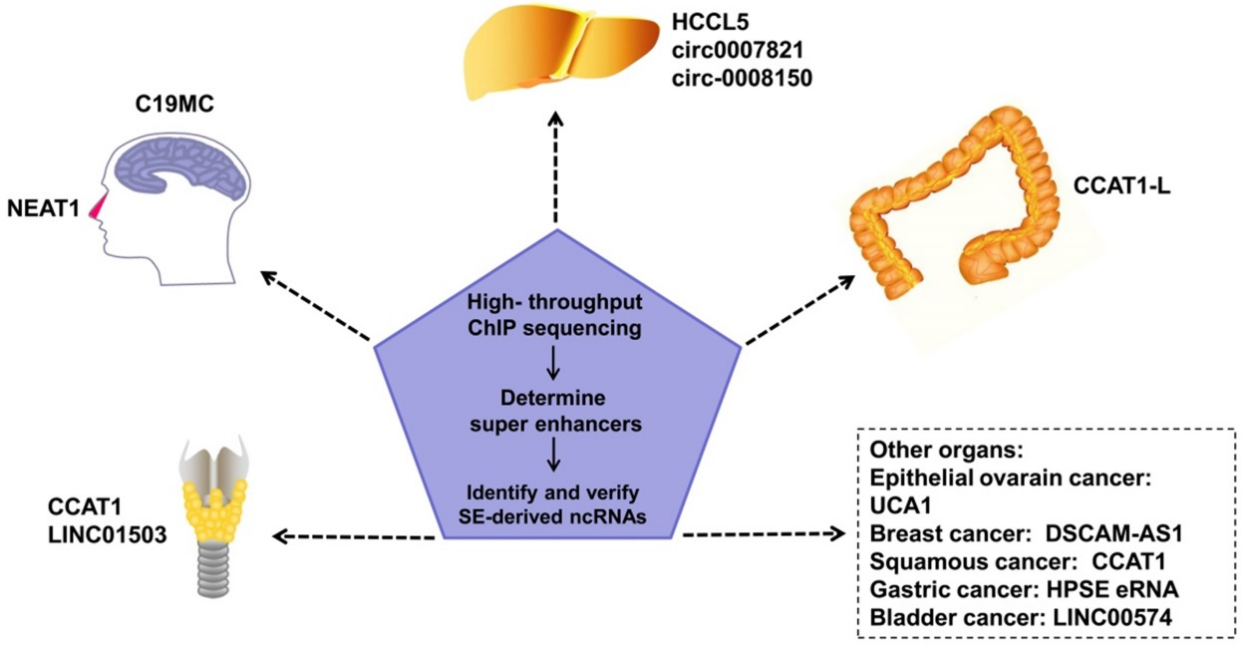
An increasing number of SE-derived ncRNAs have been reported to be aberrantly expressed in human cancers, including esophageal squamous cell carcinoma (ESCC), hepatocellular Carcinoma (HCC), epithelial ovarian cancer (EOC), embryonal tumor with multilayered rosettes (ETMRs), colorectal cancer (CRC), breast cancer, squamous cancer, lymphocytic leukemia, nasopharynx cancer.

SEs in tumor cells can drive oncogene expression by mutating to produce new SEs, chromosomal translocations of proto-SEs, local amplification, overexpression of oncogenic TFs, and other genetic mechanisms. SEs regulate the malignant biological phenotype of tumors via these mechanisms 47-49. SE-derived ncRNAs have been reported to be driven by SEs in many tumors (**Figure 2**). Estrogen receptor α in the absence of hormones (apoERα) can induce the expression of the SE-derived lncRNA DSCAM-AS1 and affect breast cancer tumorigenesis 50. There are many TNF-β-regulating SE-derived lncRNAs. Considering that hepatic stellate cells are involved in hepatic fibrosis and cirrhosis, this finding suggests that these lncRNAs are involved in liver cancer 51. In addition, regions of active enhancers or SEs can be transcribed to form eRNAs to synergize with the SEs of proto-oncogenes to further accelerate tumor occurrence and development 52. However, not all tumor-related SEs play a carcinogenic role. Some SE-derived miRNAs inhibit tumor progression 8.

### SE-derived ncRNAs, temperature regulation, and circadian rhythm

Raymond *et al.* 53 found that under cold stimulation, SE-miR-32 activates the p38/MAPK signaling pathway by inhibiting the expression of target gene Tob1 and promoting the expression of FGF21, to drive brown adipose tissue (BAT) thermogenesis and white fat browning in mice. Fan *et al.* 54 found that the SE-derived lncRNA lnc-Crot is a long-range circadian lncRNA, and its role is influenced by the TFs BMAL1 and REV-ERBα.

### SE-derived ncRNAs and immune cells

SE-derived ncRNAs are closely associated with inflammation-related diseases and immune cells. Vahedi *et al.* 55 indicated that one-third of the ncRNAs (501/1524) in T cells are formed from the self-transcription of SEs; they are known as eRNAs, and most of them have a Bach2-binding site. It is noteworthy that genetic variation at this site is associated with many immune diseases, including rheumatoid arthritis, Crohn's disease, multiple sclerosis, asthma, and type I diabetes mellitus (T1DM). Agirre *et al.* 56 reported that eRNAs from SE transcription are involved in B cell receptor activation and the humoral immune response. There is an interaction between activation-induced deaminase (AID) and SE-derived ncRNAs, which affects the production and classification of antibodies in B cells 57, 58. SE is also involved in the pathogenesis of autoimmune diseases. Many novel SE-derived lncRNAs have been found in leukocytes from patients with autoimmune diseases, such as Crohn disease and irritable bowel syndrome 59. In peripheral mononuclear erythrocytes from patients with multiple sclerosis (MS), SE enrichment and circRNA (has_circ_0043813) expression increase simultaneously 60, but the specific mechanism underlying this phenomenon has not yet been elucidated.

## Role of SE-derived ncRNAs in cancer

The article “Hallmarks of cancer: the next generation” published by Hanahan and Weinberg in 2011 in *Cell* summarizes the ten main characteristics of tumors. Recent studies have shown that SE-derived ncRNAs are closely related to the occurrence and development of tumors. SE-derived ncRNAs are involved in malignant biological behaviors such as uncontrolled proliferation, invasion and metastasis, chemoresistance, and tumor inflammatory response.

### SE-derived ncRNAs and malignant proliferation

Sustaining proliferative signaling is a fundamental feature of tumors. Normal cells tightly regulate growth-promoting and death-inducing signals to guide cell cycle and cell growth and maintain the homeostatic cell number and tissue architecture. However, these signals are deregulated in cancerous cells, thus leading to unlimited proliferation 61. The malignant proliferation of tumors involves the regulation of multiple signaling pathways (*e.g.* the PI3K pathway) and a variety of key proteins (*e.g.* cyclin).

**Figure 3:**
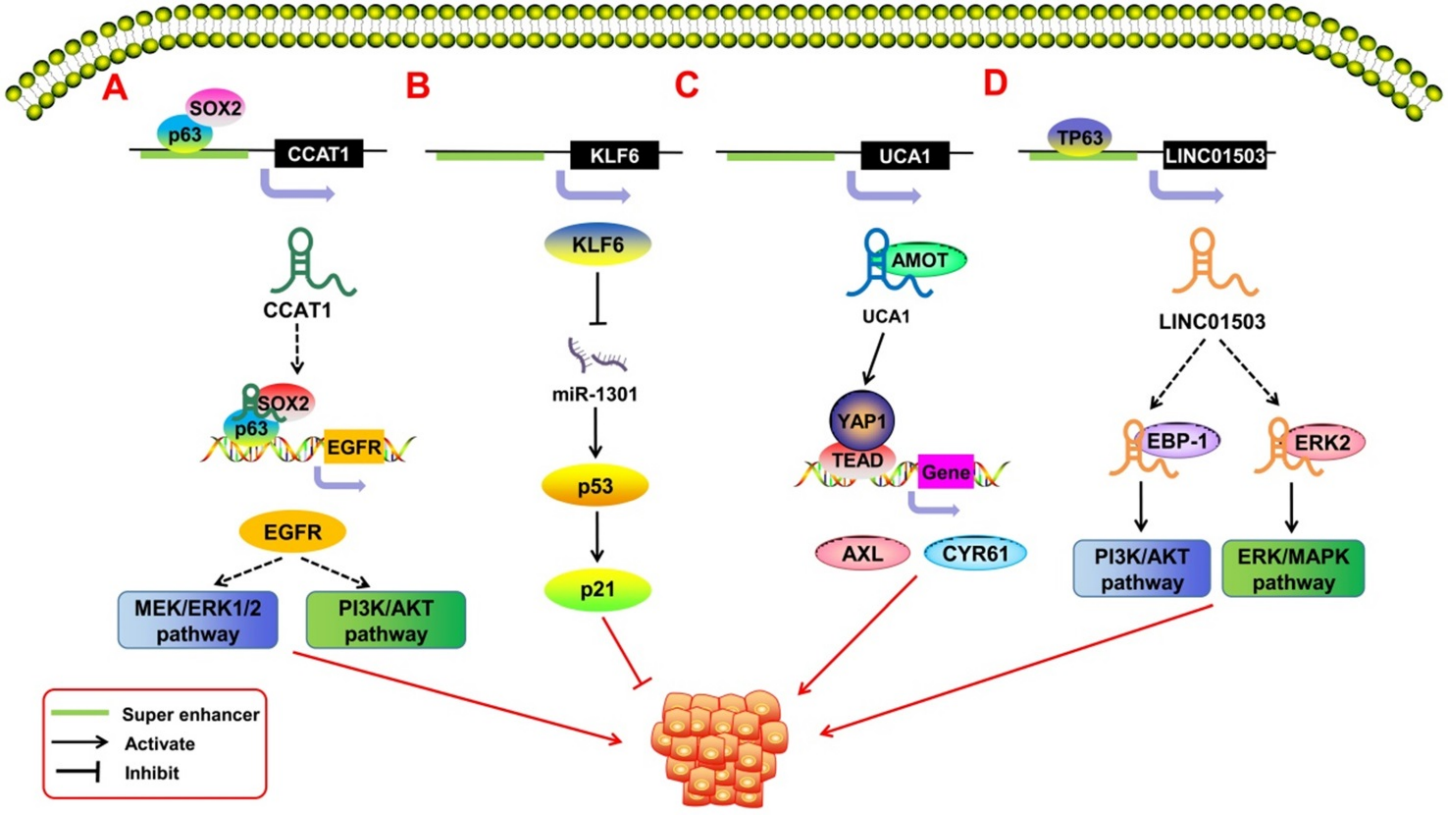
SE-derived ncRNAs regulate malignant tumor proliferation. **A.**The transcription factor SOX2 and p63 can bind to the SE region upstream of lncRNA CCAT1 to promote its expression. The obtained CCAT1 can form transcription complexes with SOX2 and p63 to regulate the expression of EGFR, leading to the malignant proliferation of SCC cells through the MEK/ERK1/2 and PI3K/AKT pathways. **B.** That KLF6 super enhancer was deleted will induce the over-expression of miR-1301, thereby inhibiting hepatoma cell proliferation by inducing p21and p53 in a p53-dependent manner. **C.** SE-derived lncRNA UCA1 interacts with AMOT to promote YAP activation and nuclear translocation and induces the expression of YAP's target genes, thereby driving the development of ovarian cancer. **D.** The transcription factor TP63 binds to the SE region upstream of LINC01503 to activate its expression; activated SE-derived LINC01503, in combination with EBP-1 and ERK2, stimulates the malignant proliferation of esophageal squamous cell carcinoma (ESCC) cells by activating the PI3K/AKT1 and ERK/MAPK signaling pathways.

In recent years, SE-derived ncRNAs have been found to play an important role in promoting the malignant proliferation of tumors (**Figure 3**). Some SE-derived ncRNAs affect proliferation-related signaling pathways, thus leading to the unlimited proliferation of tumor cells 62. Xie *et al.* 63 reported a squamous cell carcinoma (SCC)-specific SE-derived lncRNA LINC01503. The TF TP63 binds to the SE region upstream of LINC01503 in esophageal squamous cell carcinoma (ESCC) and activates it. Activated SE-LINC01503, in combination with EBP-1 and ERK2, stimulates the malignant proliferation of ESCC cells by activating the PI3K/AKT and ERK/MAPK signaling pathways. Similarly, Jiang *et al.* 64 found that in SCC, the tissue-specific lncRNA CCAT1 is regulated by SEs. CCAT1 has been shown to promote malignant proliferation in many types of tumors 6, 65, 66. SOX2 and p63 can bind to the SE region upstream of CCAT1 to regulate the transcriptional expression of CCAT1. The obtained CCAT1 can form transcription complexes with SOX2 and p63 to regulate the expression of EGFR and then lead to the malignant proliferation of SCC cells through the MEK/ERK1/2 and PI3K/AKT pathways. Xiang *et al.* 67 pointed out that SE-derived CCAT1-L can also promote tumor proliferation by enhancing the transcription of the oncogene MYC. The Hippo/YAP signaling pathway has also been associated with tumor proliferation 68, 69. Lin *et al.* 70 showed that in epithelial ovarian cancer (EOC), the SE-derived lncRNA UCA1 interacts with the Hippo/YAP1 signaling pathway and reduces the phosphorylation level of YAP1 in the cytoplasm of tumor cells. This encourages the entry of YAP1 into the nucleus and promotes the expression of the target genes *AXL* and *CYR61*, which promote cell proliferation. SE-derived ncRNAs also play an important role in virus-associated tumors. Liang *et al.* 71 showed that Epstein-Barr virus (EBV) SEs can affect the expression of the proto-oncogene MYC in the regulation of the growth and survival of lymphoblastoid cell lines, and it is an important risk factor for EBV-associated malignant proliferation.

### SE-derived ncRNAs and metastasis

Tumor metastasis is a dynamic process in which malignant cells spread to different tissues and organs from the primary tumor site and continue to proliferate and form new secondary tumors. The secondary and primary tumor tissues always have the same histological type 61, 72, 73. Tumor metastasis usually involves the following mechanisms: (1) epithelial-mesenchymal transition (EMT), (2) tumor heterogeneity and cancer stem cells, and (3) angiogenesis and hypoxia induction 74, 75.

**Figure 4:**
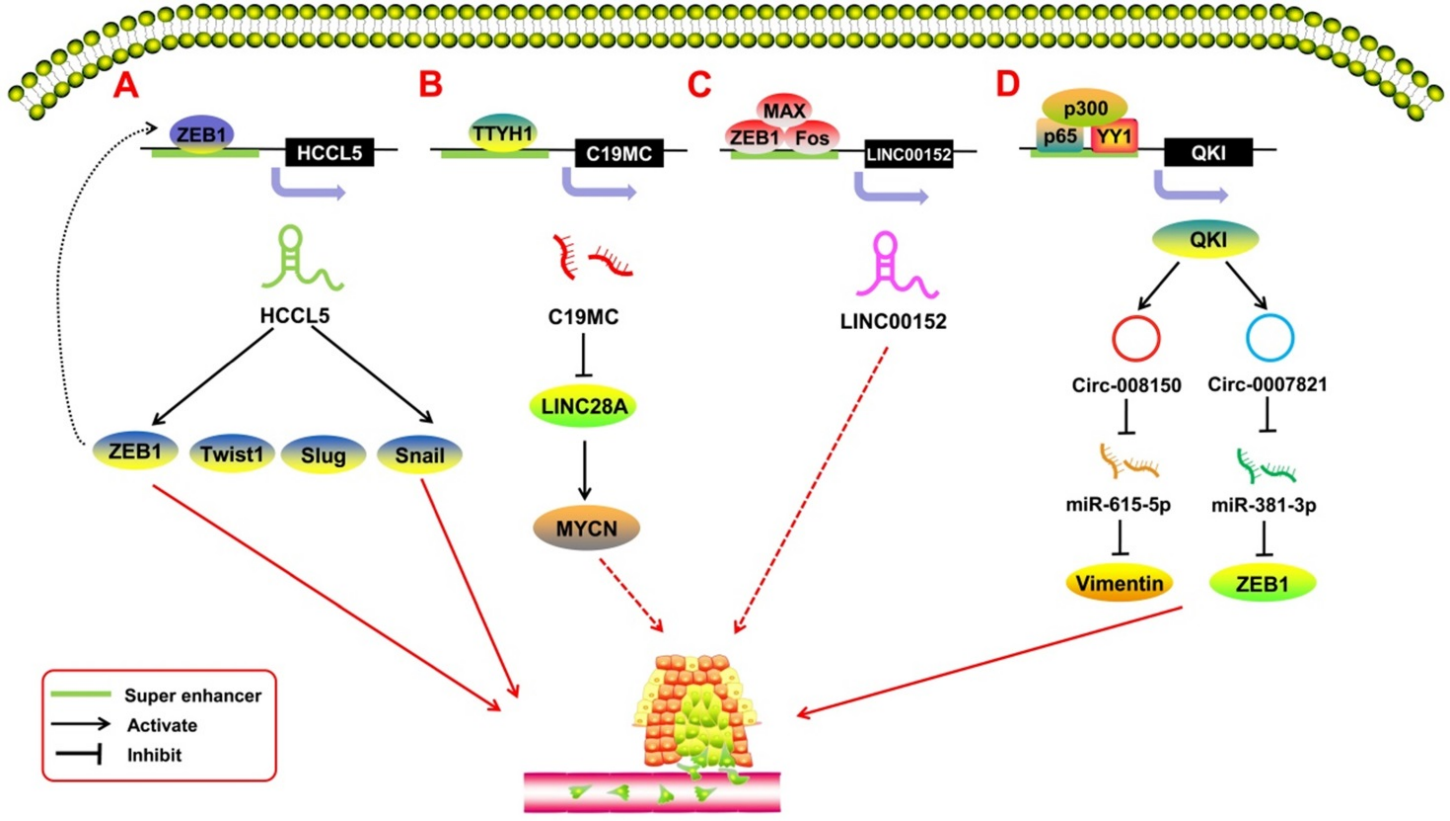
SE-derived ncRNAs regulate tumor metastasis. **A.** The transcription factor ZEB1 binds to the SE region upstream of lncRNA HCCL5 to activate its expression; activated SE-derived HCCL5 accelerates the EMT phenotype of hepatocellular carcinoma (HCC) by upregulating the expression of Snail, Slug, ZEB1, and Twist1.** B.** C19MC is overexpressed when transcription factor TTYH1 binds to the SE region and initiates the C19MC-LIN28A-MYCN circuit by inhibiting LIN28A and upregulating MYCN to regulate the progression of embryonal tumors with multilayered rosettes (ETMRs). **C.** The transcription factors FOS, ZEB1, and MAX bind to the SE region potentially upstream of the LINC00152 promoter; knocking out LINC00152 inhibits the invasion and metastasis of breast cancer cells.** D.** Transcription factor YY1 combines p65/p300 to form a transcription complex to promote quaking (QKI) expression by binding to the SE regions of QKI; meanwhile, QKI can cause the formation of circ-0008150 and circ-0007821 to promote the expression of EMT-related markers vimentin and zeb1 by adsorbing miR-615-5p and miR-381-3p.

SE-derived ncRNAs have been reported to play a crucial role in the process of tumor metastasis (**Figure 4).** EMT is the most common mechanism of tumor metastasis. Peng *et al.* 76 reported a SE-derived lncRNA, HCCL5, which is hepatocellular carcinoma (HCC)-specific. The TF ZEB1 can bind to the SE region upstream of HCCL5 to regulate its transcription. By upregulating the expression of the TFs Snail, Slug, ZEB1, and Twist1, SE-HCCL5 promotes EMT in HCC cells. Xu *et al.* 77 assessed the regulatory network of LINC00152 in pan-cancer through bioinformatics analysis and found that the TFs FOS, ZEB1, and MAX bind to the SE region potentially upstream of the LINC00152 promoter, and knocking out LINC00152 inhibits the invasion and metastasis of the breast cancer cell line MDA-MB-231. Xie *et al.* 63 indicated that SE-LINC01503 not only promotes proliferation, but also plays a positive role in SCC metastasis. Upon abolishing the expression of SE-LINC01503, the migration and invasion of SCC cells was markedly inhibited. In terms of SE-derived miRNAs, Chan *et al.* 78 reported on C19MC, a large SE-derived miRNA cluster. The SE region upstream of C19MC can be regulated by TTYH1, and overexpressed C19MC drives the C19MC-LIN28A-MYCN circuit in embryonal tumors with multilayered rosettes (ETMRs). This oncogenic circuit promotes tumor metastasis in ETMRs 79. With regard to SE-derived circRNAs, Han *et al.* 80 showed that the TF Yin-Yang 1 (YY1), combined with p65/p300, forms a transcription complex that promotes quaking (QKI) expression by binding to the SE regions of QKI. Meanwhile, QKI can cause the formation of circ-0008150 and circ-0007821 to promote the expression of EMT-related markers vimentin and zeb1 by adsorbing miR-615-5p and miR-381-3p. This process is highly activated during EMT in HCC, and the oncogenic SE-derived circRNAs would, in turn, promote the metastasis of HCC cells.

### SE-derived ncRNAs and drug resistance

Chemoresistance is the ability of tumor cells to evade or cope with the presence of chemotherapeutics and is a key challenge in cancer treatment. Chemoresistance mechanisms are complex and usually depend on tumor stem cells, apoptosis, EMT, the tumor microenvironment, and many other factors 81. The increased consumption of rapidly growing tumor cells leads to hypoxia. A hypoxic microenvironment enhances malignancy and chemoresistance of tumor cells. Hypoxia affects intracellular signaling through the TF hypoxia-inducible factor-1a (HIF-1α) which binds to the hypoxia response elements of many genes, including hexokinase and glucose transporter 1 (GLUT1). This forces the tumor cell to produce ATP through glycolysis and create a lower pH to prevent drug entry 82. Moreau *et al.* 83 reported that hypoxia can induce the activation of 358 SE regions in tumor cells, and database analysis showed that for 20% of SEs, the closest RefSeq gene was a lncRNA, as exemplified by MALAT1 and LUCAT1. MALAT1 can not only achieve chemoresistance by competitively inhibiting miR-23b-3p or miR-203, but can also bind with EZH2 to regulate CDH1 transcription expression and promote E-cadherin expression and oxaliplatin-induced EMT 84-86. Xu *et al.* 77 investigated the chemoresistance of SE-derived LINC00152 across all cancers. Through microarray technology, they found that LINC00152 may be related to methotrexate resistance in pancreatic, colon, and osteosarcoma cell lines. Yue's study 87 showed that LINC00152 sponged miR-193a-3p to block its inhibitory effect on erbB-4 and mediated the activation of the AKT signaling pathway, which is involved in tumor chemoresistance.

In addition, chemoresistance is usually associated with the inhibition of apoptosis in tumor cells. Inducing apoptosis is one of the anti-tumor mechanisms of chemotherapy drugs, while anti-apoptosis has become an effective pathway for chemoresistance 88, 89. Anti-apoptosis activity in tumors can occur in the following ways: (1) shearing pro-apoptotic genes and inhibiting TF recruitment to inactivate pro-apoptotic genes (*e.g.* p53) and (2) overexpression of anti-apoptotic genes (*e.g.* Bcl-2 and survivin) through transcriptional regulation networks 90. Studies have shown that SE-derived ncRNAs are closely related to the anti-apoptosis characteristics of tumors. Lin *et al.* 70 found that after SE-UCA1 activates the Hippo/YAP1 pathway, proliferation and cell survival are simultaneously promoted in ovarian cancer cells, showing some anti-apoptosis characteristics. In addition, Moreau *et al.* 83 found that hypoxia can regulate SE activity, which in turn, mediates tumor apoptosis, and can also activate the expression of some lncRNAs. However, whether SE regulates apoptosis by regulating the expression of lncRNAs has not been further analyzed. SE-miR-200a is a proven epithelial-specific SE-derived ncRNA 8, and miR-200a can inhibit cell apoptosis in many tumors 91-93. Therefore, we speculate that SE-miR-200a is involved in the chemoresistance mechanism whereby apoptosis is inhibited in tumor cells. To date, the mechanism underlying the action of SE-derived ncRNAs in tumor chemoresistance has not been fully elucidated, and findings obtained from high-throughput tests and bioinformatics analysis require further verification.

### SE-derived ncRNAs and inflammatory response

**Figure 5:**
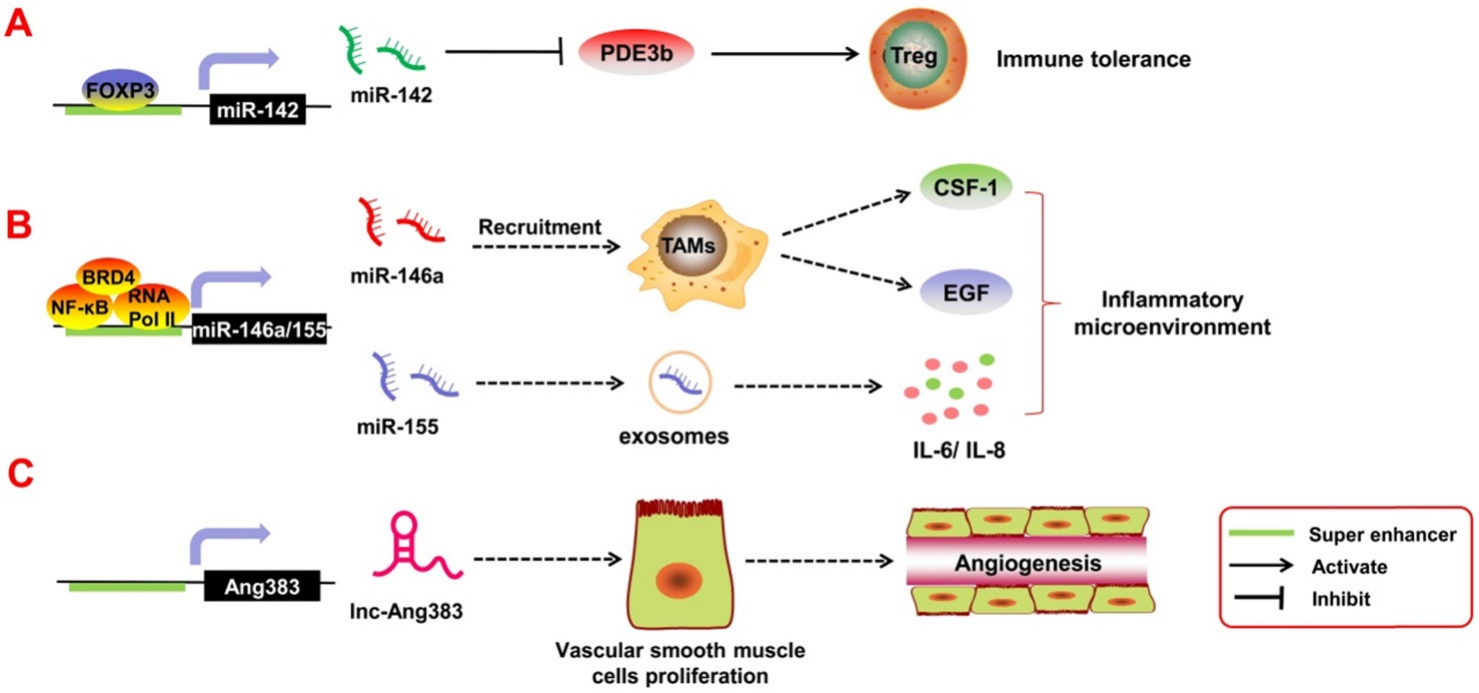
SE-derived ncRNAs regulate tumor-associated inflammatory response. **A.** The transcription factor FOXP3 can bind to the SE regions of pri-miR-142 to promote its transcription, thereby inhibiting the expression of its downstream target gene PDE3b. SE-derived miR-142 leads to immune tolerance. **B.** Transcription factors and the co-factors NF-κB, BRD4, and RNA Pol II can bind to the SE region upstream of pri-miR-146a and pri-miR-155 and promote their transcription. Tumor angiogenesis can be mediated by miR-146a, which participates in the recruitment and activation of tumor-associated macrophages to induce the secretion of epidermal growth factor (EGF) and colony stimulating factor-1 (CSF-1); miR-155 can be released into the tumor microenvironment by malignant cells and transferred into normal cells via exosomes. **C.** SE-derived lnc-Ang383 is induced to transcript in vascular smooth muscle cells (VSMCs) and promotes VSMC proliferation and angiogenesis.

Tumor inflammatory response is an important feature of tumors. Approximately 25% of tumors are caused by inflammation, and inflammatory cell infiltration occurs in almost all tumor microenvironments, and inflammatory cells and factors affect every step of tumor development 94. Recent studies reported that SE-derived ncRNAs play a crucial role in regulating tumor inflammatory response (**Figure 5**).

Immune cells such as T regulatory cells (Tregs) are important components of the tumor inflammatory microenvironment; they are involved in immune escape and immune tolerance of the tumors. If these Tregs are too active, they suppress the immune response and accelerate tumor invasion in the body. Anandagoda *et al.* 95 found that in Tregs, the TF FoxP3 can bind to the SE regions of pri-miR-142 to promote its transcription, thereby inhibiting the expression of its downstream target gene PDE3b. SE-derived miR-142 leads to immune tolerance. In addition, Duan *et al.* 96, through ChIP-seq and relevant data analysis, found that TFs and the co-factors NF-κB, BRD4, and RNA Pol II can bind to the SE region upstream of pri-miR-146a and pri-miR-155 and promote their transcription. Both miR-146a and miR-155 are inflammation-associated miRNAs. miR-146a can mediate tumor angiogenesis by participating in the recruitment and activation of tumor-associated macrophages 97. It can also promote the secretion of epidermal growth factor (EGF) and colony stimulating factor-1 (CSF-1) 98. In contrast, miR-155 can be released into the tumor microenvironment by malignant cells and transferred into normal cells via exosomes. Exosomal miR-155 regulates the expression of inflammatory factors such as IL-6 and IL-8 99. Angiotensin II (Ang II) is also an inflammatory factor in the tumor microenvironment, and it can mediate angiogenesis and tumor metastasis 100. Das *et al*. 101 reported that Ang II can induce transcription of SE-derived lnc-Ang383 in vascular smooth muscle cells (VSMCs), and it promotes VSMC proliferation and angiogenesis. However, whether Ang II/SE-derived lncRNAs can participate in tumor growth requires further exploration. Above all, it is clear that SE-derived ncRNAs play an important role in the tumor inflammatory response.

## Clinical applications of SE-derived ncRNAs in cancers

With the development of high-throughput sequencing technologies and bioinformatics prediction software, an increasing number of SE-derived ncRNAs have been identified. SE-derived ncRNAs have shown increasing significance in the early diagnosis of tumors, evaluation of tumor prognosis, targeting of therapies for tumors, and many other tumor-related clinical applications (**Table 3**).

In recent years, many studies have reported that the abnormal expression of SE-derived ncRNAs in a variety of tumor tissues is closely related to the stage, malignancy, infiltration, and other clinicopathological characteristics of patients with cancer. SE-derived ncRNAs are potential tumor markers, especially SE-derived lncRNAs. Xie *et al.* 63 reported that levels of the SE-derived lncRNA LINC01503 were substantially higher in cancerous tissues than in the adjacent non-malignant esophageal epithelium; high LINC01503 expression was significantly correlated with shorter overall survival (OS) and disease-free survival (DFS) in patients with cancer. Meanwhile, based on the Cox proportional hazard model, LINC01503 serves as an independent prognostic factor for poor survival by multivariate regression analysis. Similarly, Peng *et al.* 76 demonstrated that the expression of the SE-derived lncRNA HCCL5 was significantly higher in HCC tissues than in adjacent non-malignant tissues by ISH analysis. The expression of HCCL5 was closely related to the gender, pathological diagnosis, and tumor grade of patients with HCC; and cancer patients with high HCCL5 expression had significantly shorter OS and DFS, according to the Cancer Genome Atlas (TCGA) database analysis. Furthermore, CCAT1-L is reported to be an SE-derived lncRNA in colorectal cancer (CRC), and the expression of CCAT1-L was higher in CRC tissues than in paired normal mucosa tissues 67. Similarly, the SE-derived lncRNA NEAT1 was found to be higher in nasopharynx cancer (NPC) tissues than in the normal mucosa 104. Lin *et al.* 70 reported that the SE-derived lncRNA UCA1 was highly expressed in patients with EOC, and the expression of UCA1 was positively correlated with high-grade cancer, as determined by TCGA analysis. Therefore, UCA1 could be used as a diagnostic marker. Although some SE-derived lncRNAs have not been reported to be involved in the clinicopathological characteristics of tumors, lncRNAs have been shown to be associated with the clinicopathological characteristics of a variety of tumors in other studies. Elevated expression of SE-MALAT1 is related to TNM stage, lymph node metastasis, and poor prognosis in tumors such as lung cancer, gastric cancer, and thyroid carcinoma 105-109. SE-LINC00152 was discovered using a high-throughput pan-cancer method 77. In multiple studies and meta-analyses, the expression of LINC00152 was shown to be closely related to the clinical outcome, OS, and DFS in a variety of tumors, such as lung, liver, and stomach cancer 110-113.

As pivotal tumor markers, miRNAs and circRNAs have clinical significance in the diagnosis and prognosis of tumors such as lung cancers and colon cancers 114-116 However, there are few relevant reports on SE-derived miRNAs and SE-derived circRNAs in the clinicopathological characteristics of tumors. Patrick *et al.* 78 reported that the high expression of SE-C19MC in ETMRs was related to high malignancy and poor prognosis. Although C19MC was demonstrated to be involved in the T stage and vascular invasion in hepatocellular carcinoma 117, the clinicopathological characteristics of SE-C19MC in hepatocellular carcinoma remain unclear. MiR-155, a SE-derived miRNA 96, is overexpressed in breast cancer, cervical cancer, malignant B-cell lymphoma, and many other tumors, and the expression of miR-155 has been reported to be associated with some malignant phenotypes, such as tumor location, tumor grade, TNM staging, and distant metastasis 118-124. Derived from epithelium-specific SEs, miR-200a has also been reported in several studies to be related to the clinical characteristics and prognosis of head and neck squamous cell carcinoma and ovarian carcinoma 125-129. However, none of these studies have directly linked SE with the clinical applications of miRNAs. Thus, the clinical applications of these SE-ncRNAs deserve further studies.

## Conclusions and perspectives

SEs and ncRNAs are both hotspots in the field of tumor research, and studies have shown how SE-derived ncRNAs play a role in tumor development. SE-derived ncRNAs could become a new entry point for tumor therapeutics. Currently, there are two main types of targeted inhibitors for SE-derived ncRNAs. (1) RNA-targeting inhibitors: For example, an anti-miR-155 inhibitor and modified miR-155-DOPC liposome nanoparticles have significant inhibitory effects on tumor cells 130, 131. Furthermore, *in vivo* tumorigenesis experiments in mice showed that antisense oligonucleotides of MALAT1 could significantly inhibit tumor invasion and metastasis 132, 133. (2) SE-targeting inhibitors: The SEs inhibitors ThZ1 and JQ1 can effectively resist the expression of MYC and inhibit the proliferation, migration, and invasion of osteosarcoma cells 134. Interestingly, ThZ1 and JQ1 are broad-spectrum inhibitors; hence, whether they will remain effective towards SE-derived ncRNAs is unclear. Once demonstrated experimentally, SE inhibitors will be a new option for cancer treatment. In addition, immunotherapy has emerged as a new attractive treatment for tumors 135. An increasing number of immunosuppressors have been found; Xu *et al.* 77 demonstrated that SE-derived ncRNAs may become the next breakthrough for the immunotherapy of solid tumors.

However, there are also some limitations in the present study. First, specific SE-derived ncRNAs have different expression levels in different tumors and regulate different tumor phenotypes. This may be related to tissue specificity, but the concrete mechanism underlying this phenomenon has not been clarified. Second, databases of SE-derived ncRNAs need further improvement. There are some databases of SEs and their disease-related phenotypes 136-143 (**Table 4**), but when this article was written, there were only two databases summarizing SE-derived ncRNAs 144, 145. The database SELER established by Guo *et al.* 144 mainly includes the transcriptional regulation pathways of SE-associated lncRNAs in human tumors, and TRCirc, a circRNA database established by Tang *et al.* 145, provides resources for the efficient retrieval, browsing, and visualization of transcriptional regulation information for related sequences. The shortcomings of relevant databases will become an obstacle for future research. There is limited knowledge of SE-derived ncRNAs in clinical research. Although correlation analysis has been carried out on some SE-derived ncRNAs and tumor clinicopathological features and prognostic indicators, the guiding significance of most SE-derived ncRNAs as prognostic factors was obtained through meta-analysis and other data mining approaches, needing further clinical verification. Evidence on SE-derived ncRNAs as a therapeutic target for cancer is insufficient. Although some studies have used inhibitors to target the corresponding SE-derived ncRNAs in tumor progression experiments, most of them used animal models. The inhibitory effects of SE-derived ncRNAs in humans lack supporting data. Therefore, whether these basic research results can be translated into clinical applications remains to be elucidated.

## Figures and Tables

**Table 1:** Methods of super enhancer identification

Methods	Description	Advantages	Disadvantages	Ref.
ChIP-seq (Chromatin Immunoprecipitation sequencing)	A method for detecting genome-wide DNA segments interacting with histones and transcription factors.	1) Provides a high-resolution map of genomic expression regulation with less samples; 2) No signal noise deviations as direct sequencing method is used.	1) Unstable data accuracy as it is greatly influenced by the quality of antibodies; 2) High cost of building ChIP-seq workflow.	14, 15
3C-seq (Chromosome conformation capture)	A method for detecting the DNA-DNA interactions between enhancer regions and one other transcriptional regulatory elements.	1) Combine with quantitative PCR (qPCR) to reveal the results quantitatively; 2) No sequencing is required so the cost is low.	1) Low-throughput because interactions must be tested one at a time; 2) Not unbiased because genomic positions must be chosen to test for interactions.	16, 17
4C-seq (Circularized chromosome conformation capture)	A method for detecting genome-wide DNA-DNA interactions with a single chosen genomic location of interest.	1) Provides a high-resolution map of chromatin interactions with a chosen 'viewpoint'; 2) Fewer samples are needed for sufficient sequencing depth.	1) Inefficient because primers must be redesigned specifically before each 'viewpoint' tested; 2) Improvement is needed for data normalization and unbiased estimate.	17, 18
Hi-C (High-throughput chromosome conformation capture)	A method for detecting pairwise contacts between virtually any pair of genomic loci.	1) A matrix-balancing normalization method associated with high-resolution sequencing is developed; 2) Combines with visualization platforms to construct the 3D structure of chromatin interaction.	1) High signal noise because of polymerization state and dynamic chromatin interactions; 2) Insufficient genome-wide resolution to bridge 3D information to gene function perfectly.	19, 20
STARR-seq (self-transcribing active regulatory region sequencing)	A method to identify transcriptional enhancers and to assess their activity quantitatively by cloning DNA fragments downstream of a core promoter.	1) Provides genome-wide cell type-specific quantitative enhancer activity maps of any cell type; 2) Not affected by the location of the sequences.	Repeated identification may exist because of lack of accurate context markers.	21, 22

**Table 2:** Biological characteristics of SE-ncRNAs

SE-ncRNAs	Definition	Biogenesis mechanism	Functions	Ref.
miRNAs	MicroRNAs are small endogenous RNAs which are 19 to 25 nucleotides in size that regulate post-transcriptional gene expression.	MicroRNAs are transcribed from endogenous gene sequences to form hairpin pri-miRNAs, which are processed by Drosha/DGCR8 and further cleaved by Dicer to form mature miRNAs. SEs enhance the transcription and promote the maturity of pri-miRNAs by recruiting Drosha/DGCR8.	1) MicroRNAs can bind to the 3'-UTR region of the target mRNA and inhibit the target genes' expression at the translation level; 2) miRNAs can bind to the coding region or ORF region of the target mRNA to affect its stability; 3) miRNAs enter the nucleus and regulate the target genes' expression at the transcriptional level.	8, 30
lncRNAs	Long non-coding RNAs have a transcribing length of 200-100000 nt, lack a completely functional open reading frame (ORF), rarely encode a functional short peptide, and are located in nucleus or cytoplasm.	Five main mechanisms of lncRNA biogenesis:1) Transformation from a protein-coding gene that acquires frame disruptions; 2) Chromosome rearrangement; 3, 4) Neighboring repeats originating from two tandem duplications; 5) Insertion by a transposable element to become a functional ncRNA.	The molecular functions of lncRNAs at the epigenetic, transcriptional, and post-transcriptional levels are subdivided as follows: 1) recruiting and interacting with proteins; 2) acting as a co-regulator or a co-repressor; 3) acting as a decoy; 4) acting as host genes for miRNA; 5) interacting with miRNA.	31, 32
circRNAs	Circular RNAs are composed of >200 nucleotides and have a covalent closed loop structure without a 5' cap and/or a 3' poly (A), which can encode a small amount of polypeptide.	They are mainly produced by cyclization of exons and/or introns. They can be divided into different types, according to the method of cyclization: 1) Formation by spliceosome-dependent cable tail patching; 2) Cis-acting elements promoting formation; 3) RNA binding proteins regulating circRNA formation.	1) Circular RNAs can act as miRNA sponges. They can indirectly regulate miRNA downstream target genes' expression by preventing miRNAs from binding to the 3' untranslated regions of the mRNA; 2) They combine with RNA binding proteins (RBP), playing an important role in changing RNA splicing modes and mRNA stability; 3) They can also act as “miRNA reservoirs,” which can release large amounts of miRNAs under certain circumstances to inhibit the expression of target genes.	33-35
eRNAs	Enhancer RNA was identified as a self-transcription of the enhancer itself, with a sequence length of 0.5-5 kb.	Enhancer RNAs are transcribed from putative enhancer regions marked by histone modifications, such as H3K4m1/2 and H3K27Ac, and enriched with many transcription factors, such as LDTFs, P300, CBP, BRD4, and MED1. Recently eRNAs transcribed from super enhancers were named super-enhancer RNAs (seRNAs).	1) They synchronously combine with enhancers and promoters and enhance their interaction to stabilize the chromatin loop; 2) eRNAs initiate the transcription of targets by binding to promoters directly or indirectly via recruitment of RNA polymerase II; 3) eRNAs promote target transcription by enhancing the binding of RNA polymerase II; 4) eRNAs act as a decoy for the negative elongation factor (NELF) complex and prompt the elongation of the paused RNA polymerase II.	36, 37

**Table 3:** The correlation between SE-ncRNAs and Clinicopathological features of tumors

Tumor types	SE-ncRNA	Expression	Clinicopathology features	Ref.
Esophageal squamous cell carcinoma	LINC01503	Upregulated	Shorter overall survival and disease-free survival	63
	CCAT1	Upregulated	Lymph node metastasis and TNM staging	65
Hepatocellular carcinoma	HCCL5	Upregulated	Gender, pathological diagnosis and tumor grade, and shorter overall survival and disease-free survival	76
	circ-0008150; circ-0007821		—	80
Epithelial ovarian cancer	UCA1	Upregulated	Grade, and poorer survival	70
Embryonal tumor with multilayered rosettes	C19MC (miRNA family)	Upregulated	Frequent copy-number aberrations, and diagnosis indicator	78
Gastric cancer	HPSE eRNA	Upregulated	Local invasion, lymph node metastasis, advanced TNM stage, and shorter overall survival	102
Bladder cancer	LINC00574	Upregulated	Shorter overall survival	103
Colorectal cancer	CCAT1-L	Upregulated	—	67
Breast cancer	DSCAM- AS1	Upregulated	—	50
Squamous cancer	CCAT1	Upregulated	—	64
Lymphocytic leukemia	ESE RNA	Upregulated	—	71
Nasopharynx cancer	NEAT1	Upregulated	—	104

**Table 4:** The SE-ncRNAs associated databases

Database	Functions of database	Website	Ref.
dbSUPER	The first integrated interactive database of SEs in transcriptional regulation of cellular identities and diseases	http://bioinfo.au.tsinghua.edu.cn/dbsuper/	136
SEA	Includes SEs in multiple species and their roles in cellular identities	http://sea.edbc.org	137
SEdb	A wide range of human genome SEs and their potential roles in gene regulation	http://www.licpathway.net/sedb	138
SEanalysis	Provides a comprehensive analysis of SE-associated regulatory networks, the relationship between SE-associated genes and TFs	http://licpathway.net/SEanalysis	139
SEA version 3.0	A comprehensive extension and update of the Super-Enhancer archive.	http://sea.edbc.org	140
DEEPSEN	A convolutional neural network based method for super-enhancer prediction.	https://github.com/1991Troy/DEEPSEN	141
dbCoRC	A database of core transcriptional regulatory circuitries modeled by H3K27ac ChIP-seq signals.	http://dbcorc.cam-su.org	142
Cistrome Cancer	A comprehensive resource for predicted transcription factor (TF) targets and enhancer profiles and "super-enhancer" target genes	http://cistrome.org/CistromeCancer/	143
SELER	Transcriptional regulation of SE-lncRNAs in human tumors	http://www.seler.cn	144
TRCirc	Mainly includes transcription of circRNAs and partial SE-circRNAs	http://www.licpathway.net/TRCirc	145

## Abbreviations

SE: super enhancer
TE: typical enhancer
ncRNA: non-coding RNA
miRNA: microRNA
lncRNA: long non-coding RNA
circRNA: circular RNA
TF: transcription factor
BRD4: Bromodomain containing 4
ChIP-seq: Chromatin immunoprecipitation
eRNA: enhancer RNA
PCR: Polymerase Chain Reaction
ISH: *in situ* hybridization
mESC: mouse embryonic stem cell
apoERα: Estrogen Receptor α in absence of hormones
BAT: Brown adipose tissue
AID: Activation-induced deaminase
ESCC: esophageal squamous cell carcinoma
ESE: EBV super enhancer
LCL: lymphoblastoid cell lines
EMT: epithelial-mesenchymal
ETMRs: Embryonal tumors with multilayered rosettes
QKI: Quaking
HIF-1a: hypoxia inducible factor-1a
HRE: hypoxia responsible element
GLUT1: Glucose transporter 1
Treg: T regulatory cells
TAMs: tumor-associated macrophages
EGF: epidermal growth factor
CSF-1: colony stimulating factor-1
AngⅡ: Angiotensin Ⅱ
VSMCs: Vascular Smooth Muscle Cells
OS: overall survival
DFS: disease-free survival
ISH: *in situ* hybridization
CRC: Colorectal Cancer
ASO: antisense oligonucleotide
